# Supervisor Support, Coworker Support and Presenteeism among Healthcare Workers in China: The Mediating Role of Distributive Justice

**DOI:** 10.3390/ijerph16050817

**Published:** 2019-03-06

**Authors:** Tianan Yang, Run Lei, Xuan Jin, Yan Li, Yangyang Sun, Jianwei Deng

**Affiliations:** 1 School of Management and Economics, Beijing Institute of Technology, Beijing 100081, China; tianan.yang@bit.edu.cn (T.Y.); m18811076606@163.com (R.L.); jinxuanonly@163.com (X.J.); y.li@bit.edu.cn (Y.L.); 3120181605@bit.edu.cn (Y.S.); 2 Sustainable Development Research Institute for Economy and Society of Beijing, Beijing 100081, China

**Keywords:** supervisor support, coworker support, distributive justice, presenteeism, healthcare workers

## Abstract

Healthcare workers in China are exposed to extremely high job stress and inequitable work conditions, and the Healthy China 2030 blueprint has made them an important focus of policymakers. To examine the importance of distributive justice in Chinese medical reform, we analyzed data from 1542 healthcare workers employed in 64 primary, secondary and tertiary hospitals in 28 Chinese cities in Western, Central and Eastern China in 2018. Supervisor support, coworker support, distributive justice, and presenteeism were assessed with the supervisor support scale, coworker support scale, distributive justice scale and perceived ability to work scale, respectively. Structural equation modeling was used to examine relationships among variables. The mediating effect of distributive justice on associations between supervisor support, coworker support, and presenteeism was examined with the Sobel test. The results revealed that significant indirect effects between supervisor support and presenteeism and between coworker support and presenteeism were significantly mediated by distributive justice. Better supervisor and coworker support might improve distributive justice among healthcare workers in Chinese hospitals, thereby increasing their performance.

## 1. Introduction

Presenteeism was first conceptualized as present at work but with suboptimal performance in 1892 [1,2,3]. As its conceptualization is developing, debates on the definition of presenteeism arise. Its definition has been extended to the behavior leading to recessive lost productivity or workability attributable to health problems or other reasons [4,5,6,7], rather than only focusing on sickness presenteeism. Consequently, the perceived ability to work scale (PAWS) has been examined as a reliable and validated instrument for measuring presenteeism in this extended definition in empirical evidence [8,9] and a Nationwide survey in the U.S. Therefore, we used this definition in the current study.

Attempts to measure presenteeism have often examined costs associated with reduced work output, errors on the job, and failure to meet company production standards. Bank One (now JPMorgan Chase & Co.) estimated that presenteeism was responsible for as much as 84% of their productivity costs and that absenteeism and disability were responsible for the other 16% [10]. Presenteeism is less obvious than absenteeism, but management of presenteeism can yield important competitive advantages [1]. To control productivity loss, organizations must carefully address presenteeism behaviors.

Numerous exploratory studies have examined how potential productivity loss can be reduced by addressing presenteeism. The only interventions proven effective for presenteeism are supervisor support and coworker support [1,2,6,7], which reduce presenteeism by increasing employee satisfaction and helping workers better meet job requirements, as posited in the job demands–resources model [2,11,12]. Presenteeism can be affected by workplace psychosocial conditions such as effort–reward balance [13], which, unlike job stress, has not been frequently studied. Distributive justice—the fairness of reward allocation in an organization—is an important dimension of effort–reward balance [13]. The equity theory proposes that employees compare their effort–reward ratio to those of others [14]. When they perceive their ratio to be unfair, they may feel deprived or discontent. This psychological distress can lead to dysfunctional attitudes or behaviors, including reduced input, as a means to restore equity [14]. We hypothesized that low perceived distributive justice might decrease employees’ focus on their work, thereby increasing presenteeism. In addition, organizational justice theory holds that people’s feeling of fairness is divided into informational fairness, procedural fairness, interpersonal fairness and distributive justice. Supervisor support and coworker support help people feel respected in their organization and improve distributive justice, as supervisor and coworker support are regarded as a kind of compensation in an organization and buffer against feelings of inequity. Since supervisor and coworker support were found to affect presenteeism directly and indirectly [15,16,17,18,19], this study analyzed the mediating effect of distributive justice in the relationship between co-worker and supervisor support with presenteeism. We chose to examine this relationship among healthcare workers in China because remuneration for these workers has been a serious problem in Chinese health reform.

Although Chinese healthcare workers have low incomes, job stress is very high because of the tense relationship between physicians and patients and the considerable demands on the former [20]. Qualification requirements are stricter and training periods are longer for Chinese healthcare workers than for workers in other occupations. The relatively low incomes of healthcare workers are not commensurate with their work hours, workload or educational accomplishments. Worse still, distributive justice is low among Chinese health workers [21,22]. "Key Tasks for Deepening the Reform of the Medical and Health System in the Second Half of 2018" published by the General Office of the State Council promotes the establishment of a salary system of public hospitals that accords with the characteristics of the industry, thus highlighting the need to consider salaries of healthcare workers. 

Healthcare reform in China is mostly focused on limiting medical expenses and improving the quality of medical service [23]. Little attention has been paid to the psychological conditions of healthcare workers. Chinese healthcare workers have been victims in numerous incidents of workplace violence, and the unreasonable salary system decreases job satisfaction and diminishes enthusiasm in this population, which has resulted in poor psychological well-being among healthcare workers.

Chinese healthcare workers are essential human resources in the successful implementation of the Healthy China 2030 plan. China is a policy-driven country, and the social effects of policies are therefore much greater than in other countries [23,24]. In 2016, the Political Bureau of the Communist Party of the China Central Committee reviewed and approved the Healthy China 2030 plan, which calls for supply-side reform in order to provide better treatment to ordinary residents. Medical staff are thus required to provide an increasing number of healthcare services and to improve the quality of such services. However, the poor psychological well-being of healthcare workers in China makes them unable to meet strategic requirements, which has hampered healthcare effectiveness in the country [25,26,27]. Because of the representativeness of Chinese healthcare workers—in particular, their work conditions and their importance in Chinese medical reform—we analyzed distributive justice and the association of supervisor and coworker support with presenteeism in this population.

## 2. Materials and Methods

### 2.1. Data Source

In 2018, we surveyed 1542 healthcare workers from 64 primary, secondary and tertiary hospitals in 28 Chinese cities in Western, Central and Eastern China. Ethics approval was received from an independent research ethics committee (No. KYX2016007.) (including clinicians, nurses, administrative staff, medical technicians, and pharmacists). The survey assessed participant characteristics, supervisor support, coworker support, distributive justice, and presenteeism. To ensure data integrity and objectivity, participants were randomly selected by using employee numbers.

### 2.2. Variables and Instruments

The perceived ability to work scale (PAWS), a robust indicator of perceived productivity loss, was used to measure presenteeism. The PAWS is a reliable and valid instrument and had acceptable psychometric properties in the present study (Cronbach α = 0.829) [8,9]. Respondents were asked to rate each item from 0 (cannot currently work at all) to 10 (workability is currently at its lifetime best). To ensure that scores reflected the magnitude of presenteeism, we changed the directionality of scores by subtracting the original PAWS scores from 10. Thus, higher values indicate greater presenteeism.

Coworker support was measured with a three-item scale (five-point Likert scale: 1 = not helpful, 5 = very helpful; Cronbach α = 0.888) and supervisor support was measured with a four-item scale (five-point Likert scale: 1 = not helpful, 5 = very helpful; Cronbach α = 0.825) [25,28]. Items 1 through 4 address supervisor support, and items 5 through 7 address coworker support. Higher values reflect greater perceived support.

Distributive justice was measured by using a five-item “distributive justice scale” (five-point Likert scale: 1 = strongly disagree, 5 = strongly agree; Cronbach α = 0.912), which assesses the fairness of work outcomes and includes workload, pay level, work schedule and job responsibilities [29]. Higher scores represent better distributive justice.

To control potential confounding effects, the analysis was adjusted for sex, age, level of education, work title, seniority and duration of work experiences.

### 2.3. Data Analysis

SPSS 25.0 and AMOS 21.0 were used for statistical analysis comprising descriptive analysis and path analysis. Structural equation modeling (SEM) was used to examine relationships among supervisor support, coworker support, distributive justice, and presenteeism.

Before SEM, correlation analysis was used to determine the significance of correlations between supervisor support, coworker support, distributive justice, and presenteeism. In SEM, four latent variables—supervisor support, coworker support, distributive justice, and presenteeism—were first constructed by using the supervisor support scale, coworker support scale, distributive justice scale and PAWS indicators, respectively. The criteria used to evaluate the model were a root mean square error of approximation less than 0.08 and goodness-of-fit, normed fit, comparative fit and Tucker–Lewis index values of 0.90 or higher. All these indicators have been used to examine model fit in previous studies. The Sobel test was used to examine the effect of the mediator [30].

To determine if standardized regression coefficients (β) differed by subgroup, we analyzed participants in relation to sex, age and job title. Job title was classified as junior, intermediate and senior. Senior titles comprise deputy senior and senior job titles.

## 3. Results

### 3.1. Demographic Characteristics of Participants

Table 1 shows the demographic characteristics of the healthcare workers. Among the 1434 participants, 61.7% were women. With respect to occupation, 46.0% were clinicians, 32.6% were nurses, 6.9% were administrative staff, 10.1% were medical technicians, and 3.6% were chemists. With respect to age group, 50.2% were 25–35 years of age, and only 1.9% were older than 55 years. Data on education level showed that 46.8% had an undergraduate degree, 17.8% had a master’s degree and 17.3% had a doctorate. Nearly half the respondents (42.1%) had junior job titles, 38.1% had intermediate titles, 14.1% had deputy senior titles, and 5.7% had senior titles. Overall, 18.1% of participants had worked less than three years, 21.0% had worked 3–5 years, and 23.3% had worked 6–10 years. Internal Medicine (24.5%), Surgery (18.8%) and Obstetrics (9.6%) were the most common departmental affiliations. Only 1.9% of participants were in the Infectious Diseases/Oncology Department (Table 1).

### 3.2. Mean, SD and Correlations Between Presenteeism, Supervisor Support, Coworker Support, and Distributive Justice

The results (mean and SD) for supervisor support, coworker support, distributive justice, and presenteeism and correlations between these variables are shown by correlation coefficients (*r*) in Table 2. Presenteeism was significantly inversely correlated with supervisor support (*r* = −0.26), coworker support (*r* = −0.22) and distributive justice (*r* = −0.27). Distributive justice was significantly positively correlated with supervisor support (*r* = 0.51) and coworker support (*r* = 0.41). Supervisor support was significantly positively correlated with coworker support (*r* = 0.61).

### 3.3. SEM

**Figure 1:**
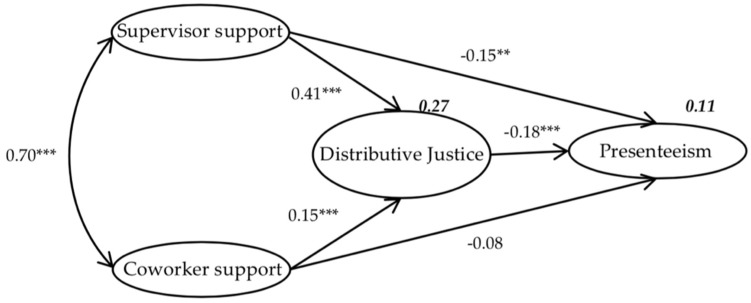
Final structural equation model, with standardized maximum likelihood estimates (numbers not in bold are standardized regression coefficients, and numbers in bold represent variability; Chi-square, 852.853, degree of freedom, 98.033, *p* < 0.001; root mean square error of approximation was 0.071, goodness-of-fit index was 0.934, comparative fit index was 0.949, Tucker–Lewis index was 0.937 and normed fit index was 0.934; ** significant at 0.001 < *p* < 0.01; *** significant at *p* < 0.001.).

Before conducting SEM, we confirmed that our model fits the data well: The goodness-of-fit index and comparative fit index values for each measurement model in the analysis of the measurement model were all between 0.936 and 0.996. The chi-squares (degree of freedom and *p*-values) for measurement model of supervisor support, distributive justice, and presenteeism were 198.418 (2, *p* < 0.001), 152.075 (5, *p* < 0.001) and 12.540 (2, *p* = 0.002). The indices of coworker support could not be computed because its measurement model was saturated. In the final SEM (Figure 1), distributive justice was directly inversely associated with presenteeism (β = −0.18, *p* < 0.001). Supervisor support was significantly inversely associated with presenteeism (β = −0.15, *p* < 0.01), but the path from coworker support to presenteeism was not significant (β = −0.08, *p* > 0.05) and was fully mediated by distributive justice. Supervisor support was directly positively associated with coworker support (β = 0.70, *p* < 0.001). Supervisor support and coworker support were significantly positively associated with distributive justice (β = 0.41, *p* < 0.001, and β = 0.15, *p* < 0.001, respectively). 

We noted significant indirect effects between supervisor support and presenteeism (Sobel *z* = −6.09; *p* < 0.001) and between coworker support and presenteeism (Sobel *z* = −6.76; *p* < 0.001), which were significantly mediated by distributive justice.

Subgroup analyses (Table 3) showed that the model results differed in relation to subgroup. For workers younger than 45 years the path from distributive justice to presenteeism (β = −0.11, *p* > 0.05) was not significant. Among female workers, workers younger than 45 years and workers with junior job titles, the path from coworker support to distributive justice was significant (β = 0.21, *p* < 0.001; β = 0.14, *p* < 0.01; and β = 0.16, *p* < 0.05). For female workers and workers with junior titles, the path from supervisor support to presenteeism was significant (β = −0.16, *p* < 0.01, and β = −0.15, *p* < 0.05, respectively).

## 4. Discussion

Supervisor support and coworker support had different effects on distributive justice and presenteeism. Supervisor support had a significant effect on presenteeism, but coworker support did not. Although coworker support had a significant effect on distributive justice, supervisor support was even more effective in improving distributive justice. Distributive justice fully mediated the relationship between coworker support and presenteeism and partially mediated the relationship between supervisor support and presenteeism. In this study, coworker support and supervisor support explained 22% of the variability in distributive justice and 17% of the variability in presenteeism. 

An interesting finding of this study is that distributive justice directly affected presenteeism while mediating the relationship between supervisor support, coworker support, and presenteeism. To our knowledge, this is the first study to examine the direct influence of distributive justice on presenteeism and to report the mediating effects of distributive justice. Our findings show that enhancing distributive justice reduced presenteeism among Chinese healthcare workers while increasing support from supervisors and coworkers. Chinese health reforms have mainly focused on limiting medical expenses and improving the quality of medical services [23] and have offered little in regard to the mental health and psychosocial concerns of healthcare workers. Therefore, in the new round of Chinese health reform, further efforts regarding enhancement of distributive justice, such as a flexible, diverse salary distribution for healthcare workers, and reasonable prices for healthcare services, should be considered in order to control hospital costs.

An interesting finding of this study is that supervisor support directly affected presenteeism. Mintzberg, in “The Nature of Managerial Work”, holds that managers have 10 roles, including their roles as corporate spokespersons, information disseminators, and resource distributors. Compared with colleagues, supervisors can mobilize more resources to help subordinates, and supervisor help is more readily available for healthcare workers. In this situation, extensive supervisor support is likely to improve productivity among healthcare workers. 

Subgroup analysis yielded varied results, the most interesting of which were that only the path from supervisor support to distributive justice and the path from supervisor support to presenteeism were significant for workers older than 45 years. Therefore, because distributive justice does not affect presenteeism, managers can focus exclusively on enhancing supervisor support, rather than on distributive justice, when combatting presenteeism among workers older than 45 years. For male workers and workers with intermediate and senior job titles, only supervisor support affected distributive justice, and distributive justice directly affected presenteeism. For these worker subgroups, it might be possible to reduce presenteeism by promoting supervisor support, thus improving distributive justice. Our results for female workers, workers younger than 45 years and workers with junior job titles are consistent with those for the overall population. Interventions, such as improved supervisor and coworker support, and a greater focus on the extent of distributive justice in the workplace, could reduce presenteeism and promote productivity.

Our findings accord with those of some previous studies. We confirmed that supervisor and coworker support helped promote productivity [2,29,30]. In addition, strong supervisor support and coworker support were associated with higher productivity and less presenteeism [30,31,32]. A future study should examine the mediating effects of job stress on the relationship between distributive justice and presenteeism.

The present findings have several theoretical and practical benefits. From a theoretical perspective, our findings broaden the concept of presenteeism and provide empirical evidence for further investigations of interventions for presenteeism. To increase awareness of psychosocial factors in healthcare management, we investigated the impact of psychosocial factors on presenteeism. With respect to the practical implications for healthcare management, we have provided empirical evidence—from a psychosocial perspective—that should assist in controlling the significant potential costs for hospitals. Specifically, our analysis of the mediating effects of distributive justice between supervisor support, coworker support, and presenteeism provides evidence for salary reform as part of healthcare policy and suggests that policymakers need to be more mindful of the psychological condition of healthcare workers. On the other hand, our findings show that appropriate supervisor support and coworker support could increase distributive justice. This implies that managers could consider enhancing supervisor support and create a supportive work climate to improve perceived distributive justice of employees.

This study has four limitations. First, the analysis focused narrowly on productivity loss rather than on productivity gain in relation to absenteeism. Second, the findings of this cross-sectional study require confirmation in a cohort study. Third, our use of self-reported presenteeism rather than quantitative measures limits the generalizability of our conclusions. Fourth, some factors that have important effects on presenteeism, such as workplace policies, were not investigated. 

## 5. Conclusions

Achieving Healthy China goals will require greater attention to the physical and psychological condition of healthcare workers in China. Such workers have prohibitively high job stress and are employed in hospitals with poor distributive justice. Our findings suggest that salary system reform could be an essential aspect of Chinese medical reform. In addition, the presence of adequate coworker and supervisor support enhances distributive justice and reduces presenteeism, which are vitally important for medical reform and efforts to cope with challenges facing Chinese hospitals.

## Figures and Tables

**Table 1:** Demographic characteristics of the participants.

	Final Sample (*n* = 1434)	Percentage (%)
Sex		
Male	572	38.3
Female	923	61.7
Age, years		
<25	126	8.3
25~30	412	27.1
31~35	351	23.1
36~40	216	14.2
41~45	154	10.1
46~50	150	9.9
51~55	83	5.5
56~60	28	1.8
>60	1	0.1
Position		
Clinician	627	46.0
Nurse	445	32.6
Administrative staff	94	6.9
Medical technician	138	10.1
Chemist	49	3.6
Other	11	0.8
Education		
Less than junior college	75	5.0
Junior college	351	23.2
Bachelor’s degree	708	46.8
Master’s degree	270	17.8
Doctoral degree	110	7.3
Job Title		
Junior	615	42.1
Intermediate	556	38.1
Deputy senior	206	14.1
Senior	83	5.7
Duration of employment, years		
<3	272	18.1
3~5	317	21.0
6~10	351	23.3
11~20	293	19.5
>20	273	18.1
Department		
Internal Medicine	367	24.5
Surgery	282	18.8
Obstetrics	144	9.6
Pediatrics	127	8.5
Chinese Medicine/ Rehabilitation	62	4.1
Emergency/Intensive Care Unit	72	5.3
Infectious Diseases/Oncology	29	1.9
Other clinical departments	98	6.5
Medical technicians	133	8.9
Administration and logistics	88	5.9
Other	88	5.9

**Table 2:** Intercorrelations between presenteeism (P), supervisor support (SS), coworker support (CS) and distributive justice (DJ) items.

Variables (Mean, SD)	Items			
	P	SS	CS	DJ
*P* (3.38, 1.28)	1	-	-	-
*SS* (3.54, 0.78)	−0.26 **	1	-	-
*CS* (3.80, 0.69)	−0.22 **	0.61 **	1	-
*DJ* (3.19, 0.83)	−0.27 **	0.51 **	0.41 **	1

** *p* < 0.01; SS, supervisor support; CS, coworker support; DJ, distributive justice; P, presenteeism.

**Table 3:** Standardized regression weights (β) with p-values (α = 0.05) for the components of subgroup analyses.

Path	Sex				Age, years				Title					
	Male		Female		<45		>45		Junior		Intermediate		Senior	
	β	*p*	β	*p*	β	*p*	β	*p*	β	*p*	β	*p*	β	*p*
*SS* to *DJ*	0.50	***	0.34	***	0.41	***	0.43	***	0.4	***	0.4	***	0.47	***
*CS* to *DJ*	0.06	-	0.21	***	0.14	**	0.16	-	0.16	*	0.09	-	0.14	-
*DJ* to *P*	−0.18	**	−0.18	***	−0.19	***	−0.11	-	−0.21	***	−0.14	*	−0.19	*
*SS* to *P*	−0.11	-	−0.16	**	−0.12	*	−0.31	*	−0.15	*	−0.14	-	−0.1	-
*CS* to *P*	−0.15	-	−0.03	-	−0.08	-	−0.02	-	−0.08	-	−0.21	-	−0.16	-

SS, supervisor support; CS, coworker support; DJ, distributive justice; P, presenteeism. * Significant at 0.01 < *p* < 0.05; ** significant at 0.001 < *p* < 0.01; *** significant at *p* < 0.001. A hyphen (-) indicates that the path is not significant.

## References

[1] Johns G. (2010). Presenteeism in the workplace: A review and research agenda. J. Organ. Behav..

[2] Yang T., Shen Y.M., Zhu M., Liu Y., Deng J., Qian C., See L.C. (2015). Effects of Co-Worker and Supervisor Support on Job Stress and Presenteeism in an Aging Workforce: A Structural Equation Modelling Approach. Int. J. Environ. Res. Public Health.

[3] Yang T., Guo Y., Ma M., Li Y., Tian H., Deng J. (2017). Job Stress and Presenteeism among Chinese Healthcare Workers: The Mediating Effects of Affective Commitment. Int. J. Environ. Res. Public Health.

[4] Goetzel R.Z., Long S.R., Ozminkowski R.J., Kevin H., Shaohung W., Wendy L. (2004). Health, absence, disability, and presenteeism cost estimates of certain physical and mental health conditions affecting U.S. employers. J. Occup. Environ. Med..

[5] Bergstrom G., Lhagberg B. (2009). Does sickness presenteeism have an impact on future general health?. Int. Arch. Occup. Environ. Health.

[6] Yang T., Zhu M., Xie X. (2016). The determinants of presenteeism: a comprehensive investigation of stress-related factors at work, health, and individual factors among the aging workforce. J. Occup. Health.

[7] Yang T., Ma M., Zhu M., Liu Y., Chen Q., Zhang S., Deng J. (2018). Challenge or hindrance: Does job stress affect presenteeism among Chinese healthcare workers?. J. Occup. Health.

[8] Ilmarinen J., Rantanen J. (2010). Promotion of work ability during ageing. Am. J. Ind. Med..

[9] Kimmo V., Virtanen P., Luukkaala T., Nygård C.H. (2012). Relationship between perceived work ability and productivity loss. Int. J. Occup. Saf. Ergon..

[10] Paul H. (2004). Presenteeism: At work—But out of it. Harvard Bus. Rev..

[11] Pritchard R.D., Karasick B.W. (1973). The effects of organizational climate on managerial job performance and job satisfaction. Organ. Behav. Hum. Perform..

[12] Coffeng J.K., Hendriksen I.J.M., Duijts S.F.A., Twisk J.W.R., Willem V.M., Boot C.R.L. (2014). Effectiveness of a combined social and physical environmental intervention on presenteeism, absenteeism, work performance, and work engagement in office employees. J. Occup. Environ. Med..

[13] Saijo Y., Yoshioka E., Nakagi Y., Kawanishi Y., Hanley S.J.B., Yoshida T. (2017). Social support and its interrelationships with demand–control model factors on presenteeism and absenteeism in Japanese civil servants. Int. Arch. Occup. Environ. Health.

[14] Pritchard R.D. (1969). Equity theory: A review and critique. Organ. Behav. Hum. Perform..

[15] Tepper B.J. (2001). Health Consequences of Organizational Injustice: Tests of Main and Interactive Effects. Organ. Behav. Hum. Decis. Process..

[16] Elovainio M., Den Bos K.V., Linna A., Kivimaki M., Alamursula L., Pentti J., Vahtera J. (2005). Combined effects of uncertainty and organizational justice on employee health: Testing the uncertainty management model of fairness judgments among Finnish public sector employees. Soc. Sci. Med..

[17] Moliner C., Martineztur V., Peiro J.M., Ramos J., Cropanzano R. (2005). Relationships Between Organizational Justice and Burnout at the Work-Unit Level. Int. J. Stress Manag..

[18] Choi B.K., Moon H.K., Nae E.Y., Ko W. (2013). Distributive justice, job stress, and turnover intention: Cross-level effects of empowerment climate in work groups. J. Manag. Organ..

[19] Maslach C., Schaufeli W.B., Leiter M.P. (2001). Job Burnout. Ann. Rev. Psychol..

[20] Mccue J.D. (1982). The Effects of Stress on Physicians and Their Medical Practice. N. Engl. J. Med..

[21] Lin L., Li H., Zhang X. (2015). Attitude and Cause Analysis of Chinese Medical Staffs on Salary Fairness in 45 Hospitals of 9 Provinces. Chin. Hosp. Manag..

[22] Adams J.S. (1965). Inequity In Social Exchange. Adv. Exp. Soc. Psychol..

[23] Ogińska-Bulik N. (2006). Occupational Stress and Its Consequences in Healthcare Professionals: The Role of Type D Personality. Int. J. Occup. Med. Environ. Health.

[24] Liu G., Liu X., Meng Q. (1994). Privatization of the medical market in socialist China: A historical approach. Health Policy.

[25] Haynes C.E., Wall T.D., Bolden R.I., Stride C., Rick J.E. (2011). Measures of perceived work characteristics for health services research: Test of a measurement model and normative data. Br. J. Health Psychol..

[26] Blau G.J. (2011). The measurement and prediction of career commitment. J. Occup. Organ. Psychol..

[27] Li L., Fu H. (2017). China’s health care system reform: Progress and prospects. Int. J. Health Plan. Manag..

[28] Robert E., Florence S., Christian V., Sucharski I.L., Linda R. (2002). Perceived supervisor support: Contributions to perceived organizational support and employee retention. J. Appl. Psychol..

[29] Niehoff B.P., Moorman R.H. (1993). Justice as a Mediator of the Relationship Between Methods of Monitoring and Organizational Citizenship Behavior. Acad. Manag. J..

[30] Viswesvaran C., Sanchez J.I., Fisher J. (1999). The Role of Social Support in the Process of Work Stress: A Meta-Analysis. J. Vocat. Behav..

[31] Brown S.L., Nesse R.M., Smith V.D.M. (2003). Providing Social Support May Be More Beneficial than Receiving It: Results from a Prospective Study of Mortality. Psychol. Sci..

[32] Poulsen M., Khan A., Poulsen E.E., Khan S.R., Poulsen A.A. (2016). Work engagement in cancer care: The power of co-worker and supervisor support. Eur. J. Oncol. Nurs..

